# High-Pressure Study of Anatase TiO_2_

**DOI:** 10.3390/ma3031509

**Published:** 2010-03-01

**Authors:** Jaćim Jaćimović, Cristian Vâju, Richard Gaál, Arnaud Magrez, Helmuth Berger, László Forró

**Affiliations:** EPFL, School of Basic Sciences, Station 3, CH-1015 Lausanne, Switzerland; E-Mails: cristian.vaju@epfl.ch (C.V.); richard.gaal@epfl.ch (R.G.); arnaud.margez@epfl.ch (A.M.); helmuth.berger@epfl.ch (H.B.); laszlo.forro@epfl.ch (L.F.)

**Keywords:** transparent semiconductor, resistivity, thermo-electric power

## Abstract

We report resistivity and thermo-electric power measurements of the anatase phase of TiO_2_ under pressure up to 2.3 GPa. Despite its transparent appearance, the single crystal of anatase exhibits a metallic-like resistivity above 60 K, at all pressures. The rather high value of the thermo-electric power at room temperature points to complex transport mechanism in this phase.

## 1. Introduction

For several decades, huge effort has been devoted to study transparent conducting oxides due to their potential applications in optoelectronic devices (such as flat panel displays, organic light emitting displays), and especially in dye sensitized solar cells [1]. Furthermore, TiO_2_ was found to be an efficient photocatalyst and a good candidate for low-cost production of solar cells [2].

There are three distinct forms of TiO_2_ in nature: rutile, anatase, and brookite. The rutile phase (P4_2_/*mnm* space group, $D_{4h}^{14}$) is the most stable one, therefore the electronic and optical properties of it have been well explored. The brookite phase (P*bca* space group, $D_{2h}^{15}$) occurs quite rarely, whilst the anatase phase (I4_1_/*amd* space group, $D_{4h}^{19}$) is unstable at elevated temperatures. The electronic properties of the latter two $D_{4h}^{19}$) are still largely unexplored. The crystal structure of undoped anatase is tetragonal with lattice parameters a = 3.782Å and c = 9.502Å [3] and has a semiconductor band gap of 3.2 eV [4]. Titanium is a transition metal with the normal atomic configuration of (4s)^2^(3d)^2^ outside the argon core [5]. The oxidation state of oxygen is -2, and the normal atomic configuration is (1s)^2^(2s)^2^(2p)^4^. This fact leads to the s-p-d hybridization determining the final structure of TiO_2_. The structure of anatase can be considered as built up of slightly distorted TiO_2_ octahedra. Even though transport properties at ambient pressure have been reported earlier [6], no information concerning transport properties under pressure has been published yet, although this information would be useful for detailed analyses of the conduction process. Our goal is to use pressure and temperature as control parameters in order to investigate in details their effect on electron mobility, and in particular the role of electron-phonon coupling on the electronic transport.

Two transport coefficients have been measured: resistivity and thermo-electric power (S). Both properties have been investigated as a function of temperature in the 1.5–300 K range, at several pressures between 0–2.3 GPa.

## 2. Experimental Section

In our laboratory we have succeeded to prepare large bluish transparent single crystals of anatase TiO_2_ that allowed us to study its bulk electronic properties. The anatase crystals were grown by chemical transport method as described in reference [6]. The samples were annealed in air at 600 ºC causing the disappearance of the bluish hue and leaving colorless sample.

The electrical resistivity and thermo-electric power have been measured up to 2.3 GPa using a piston-cylinder high pressure cell in a wide temperature range. The sample was cut into a rectangular bar (typical dimensions 0.8 × 0.6 × 0.15 mm^3^), placed horizontally on a ceramic holder, and contacted with silver paste on pre-deposited gold pads. This configuration allowed simultaneous measurement of resistivity (four probe), and thermo-electric power. For the S measurement, a small metallic heater was installed at one end of the ceramic holder, which generated a temperature gradient, measured by a calibrated chromel/constantan differential thermocouple. The pressure transmission medium used in this study was Daphne-oil.

## 3. Results and Discussion

### 3.1. Resistivity properties

**Figure 1:**
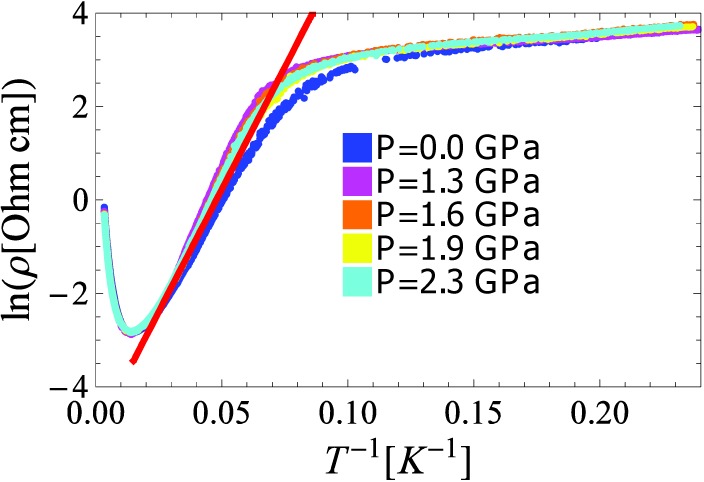
ln(ρ) versus 1/*T* of an anatase single crystal at various pressures. Above 60 K the sample is in the exhaustion regime, with resistivity having a positive slope, whilst below 60 K the conduction is activated. The red line is a fit to the activated behavior, as described in the text.

In Figure 1, the resistivity is plotted as a function of 1/T and it reveals two distinct regimes:

(i) Above 60 K, surprisingly we observe that the resistivity increases with temperature in a metal-like fashion, despite the transparent look of the sample. Although the temperature dependence is metallic, the rather high absolute value of the resistivity (0.8 Ωcm), much above the Ioffe-Regel limit for metallic resistivity, suggests an unconventional transport mechanism. Below 60 K, the resistivity rises with decreasing temperature.

**Figure 2:**
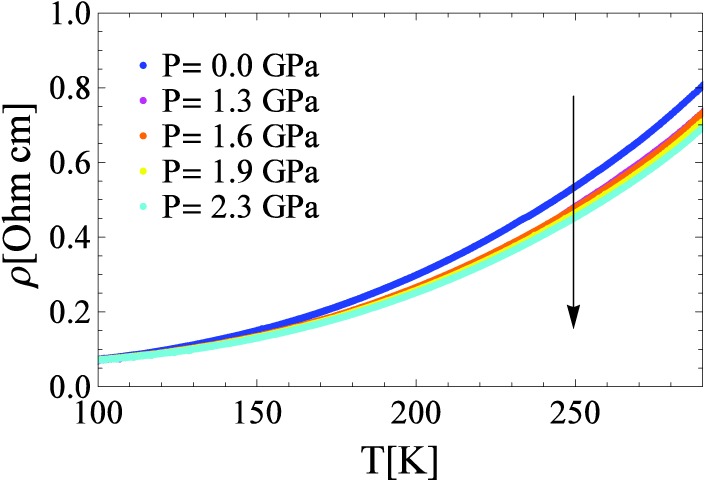
The temperature dependence of the resistivity in the exhaustion regime (above 100 K) at various pressures.

In order to elucidate the electronic state at intermediate temperatures, in the range of 60 to 20 K, we have fitted the results presented in Figure 1, and found that a thermally activated behavior describes it reasonably well. Mathematically it is modeled by:

(1) $$\rho \left(T\right)=\rho_{0}e^{\Delta /k_{B}T}$$

where $\rho_{0}$ is only slightly temperature dependent and can thus be regarded as a constant in the given temperature range, $k_{B}$ is the Boltzman’s constant, and Δ is the activation energy for charge transport. It has been shown [6] that the electron mobility in the metal-like temperature regime tends to saturate as temperature approaches 60 K. In our single band model fit, we assume that the saturation trend is extended to lower temperatures. The slope of the ln(ρ) *vs.* 1/*T* at ambient pressure yields 8 meV for the activation energy. We ascribe this low energy value to a shallow donor level, possibly coming from oxygen vacancies. The motivation for this assumption we find in earlier reports, where it has been shown that pure TiO_2_ is an insulator with 3.2 eV gap; hence the presence of ions of the same element with different valence at crystallographically equivalent sites can be a major source of carriers [7]. In the anatase n-type doping can be achieved by creating oxygen vacancies. Upon further decrease of temperature from 20 to 1.5 K a change of the resistivity slope is observed, retaining the activated temperature dependence. This indicates the presence of an even shallower donor level. The calculated activation energy in the low temperature domain is 0.8 meV. This donor level might have similar origin like the previous one, or alternatively it might originate from nitrogen, which could be captured during synthesis and/or the annealing of the sample [8]. The low temperature activation energy is pressure independent up to 2.3 GPa. Even at the lowest temperature in this study, the conductivity of the sample remained relatively high, raising the possibility of hopping conduction.

(ii) Figure 2 shows the high temperature part of the resistivity curves on a linear scale. Since over the whole range (100 K–300 K) the temperature is significantly larger than the ionization energy of the donor levels, the carrier concentration does not change within this interval, and the temperature dependence of the resistivity is dominated by the temperature dependence of the relaxation rate, giving rise to the metallic-like behavior. The resistivity decreases strongly with applied pressure; at 2.3 GPa its room temperature value drops by 15%. We note that above 100 K the resistivity curves for all the pressures can be scaled onto each other. This indicates that the scattering process is not significantly influenced by pressure. The low absolute value of the conductivity can be explained by heavy carriers. The large effective mass of charge carriers might come from the narrowness of the d-bands and it could be due to polaronic enhancement. We have found that in the 100–300 K temperature range the measured resistivity can be reasonably well described by two terms: electron-electron interaction, and a Bloch-Grüneisen term describing electron-phonon coupling. Within this model, the decrease of resistivity with pressure would follow naturally. However, we note that consistent application of simple scattering theory requires the carrier’s mean-free path to be at least as long as the lattice constant. The rather high resistivity at room temperature argues that this condition may not be fulfilled. Therefore, the puzzling resistivity properties in the exhaustion regime are subject of future studies.

### 3.2. Thermo-electric power

**Figure 3:**
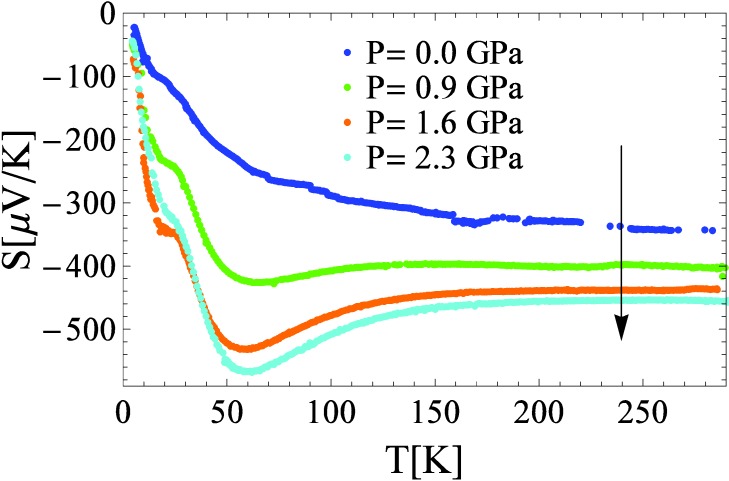
Temperature dependence of the thermo-electric power shown at various pressures.

It is interesting to see how pressure influences another transport coefficient, the thermo-electric power, called also as Seebeck coefficient (S). Figure 3 shows the temperature dependence of the S from ambient pressure up to 2.3 GPa. It is known that this quantity filters better the electronic processes at the Fermi surface than resistivity does. The pressure dependence of the S sheds additional light on the possible mechanism of conductivity.

It is known that the dominant type of carriers present in the specimen determines the sign of the thermo-electric power. In our case, the negative sign of the Seebeck coefficient indicates electron-like charge carriers. This justifies the identification of the dopants as donors in the discussion concerning resistivity. Although S decreases with temperature like in metals, its high value at room-temperature (~300 μV/K) and its non-linear temperature dependence indicate a complex charge transport mechanism. The overall shape and the magnitude of the curve resemble those of boron carbides measured by Aselage and co-workers [9]. In that system thermo-electric power is believed to be enhanced by polaronic effects, which are present despite the high concentration of carriers [10]. It is worth mentioning that the obtained temperature dependence of S at ambient pressure does not have the same behavior as it is reported earlier [6]. One explanation is already given in reference [6], and that is the possible anisotropy of the thermo-electric power.

The applied pressure increases the room-temperature absolute value of S from 300 to 450 µV/K. Pressure induces two anomalies in thermo-power behavior, a broad peak around 60 K, and a smaller shoulder at ~20 K. Both appear at temperature values where resistivity shows a change in slope.

The explanation evoked by Emin to explain the enhanced thermo-power of boron carbides in [10] was the softening of vibrational modes by the localized carriers. Along his ideas, the thermal localization of the carriers might lead also in TiO_2_ to a local softening and concomitant enhancement of the thermo-electric power.

## 4. Conclusions

In summary, we have measured the resistivity and thermo-electric power of anatase TiO_2_ as a function of temperature under various pressures. The resistivity can be modeled by a doped semiconductor with two different shallow donor levels having activation energies around 10 K and 100 K. This leads to metallic-like temperature dependence of the resistivity above 100 K. Unlike resistivity, thermo-electric power, both in temperature dependence and absolute value, resembles the one of boron carbide, suggesting strong polaronic effects in our compound, as well. Surprisingly, pressure increases the absolute value of thermoelectric power, which develops two peaks at low temperatures where the carrier concentration varies strongly. The understanding of these phenomena might be important for the understanding of polaronic transport in general, and will be the subject of future studies.
